# A latent profile analysis of doctors’ joy in work at public hospitals

**DOI:** 10.3389/fpsyg.2024.1330078

**Published:** 2024-03-21

**Authors:** Weilin Zhu, Jiayi Li, Liqun Wu, Fang Du, Yi Zhou, Kaichuan Diao, Huatang Zeng

**Affiliations:** ^1^Shenzhen Health Development Research and Data Management Center, Shenzhen, China; ^2^Wuhan Children’s Hospital (Wuhan Maternal and Child Healthcare Hospital), Tongji Medical College, Huazhong University of Science and Technology, Wuhan, Hubei, China

**Keywords:** public hospitals, doctors, joy in work, latent profile analysis, work stress

## Abstract

**Introduction:**

When doctors’ work stress increases, their joy in work decreases, severely affecting the quality of care and threatening patient safety. Analysis of the latent categories of joy in work of doctors in public hospitals and the differences in the characteristics of each category can help uncover hidden messages that enhance doctors’ joy in work.

**Methods:**

Questionnaires were administered to 426 doctors working in public hospitals using the general information questionnaire and the public hospital doctor’s joy in work evaluation scale. Upon identifying their potential categories using latent profile analysis, chi-square test, and multinomial logistic regression were performed to analyze the differences in the characteristics of each category.

**Results:**

The 426 public hospital doctors could be divided into three potential categories: “low joy in work” (11.27%), “medium joy in work” (59.86%), and “high joy in work” (28.87%). Most of the doctors did not have much joy in work, with 71.13% of them having “low to medium joy in work.” Doctors who work in secondary or tertiary hospitals, have a personnel agency or contract, and are older than 45 years are more likely to belong to the “low joy in work” category. Some of the protective factors are having an average monthly income (RMB) of 10,001–15,000 yuan and having a fair or good self-rated health status.

**Conclusion:**

There are obvious classification characteristics of doctors’ level of joy in work. Hospital managers can take commensurate actions to improve their joy in work, thereby improving patient safety and the quality of medical services.

## 1 Introduction

Joy in work was first proposed as a concept in the 1980s and has been subjected to extensive investigation in areas such as management and psychology (Deal and Kennedy, 1983; Everett, 2011). While the medical field emphasizes the medical staff’s experience of practice and work environment, joy in work is beginning to receive attention as an innovative managerial perspective. Joy in work is defined as the individual’s intellectual, behavioral, and emotional commitment to meaningful and satisfying work, as well as the sense of achievement and success experienced in the workplace (Sirota et al., 2005; Sikka et al., 2015).

Although the world has past the COVID-19 pandemic’s most challenging period of attack, lingering effects on healthcare workers continue to persist. Related research shows a dramatic increase in healthcare stress and distress during COVID-19. As the pandemic intensified, anxiety, depression, and a decrease in work joy increased, severely threatening the physical and mental safety of both doctors and patients (Luo et al., 2020; Ornell et al., 2020; Spoorthy et al., 2020). Therefore, how to improve doctors’ joy in work and reduce the negative effect on it has become an important focus of research. High levels of joy in work can diminish burnout, anxiety, depression, and other negative emotions among medical staff, as well as enhance motivation and initiative, thereby improving patient safety and quality of care (Sikka et al., 2015; Resource and Perlo, 2017; Zhang et al., 2021).

Currently, COVID-19 has been effectively controlled around the world, with many countries adopting an open-door policy. In China, the COVID-19 pandemic has progressed to the stage of standardized open-ended management, and strong control measures taken prior to liberalization have prevented the wide spread of the virus, which cannot be accomplished without strong policy protection and selfless devotion to medical and nursing staff. Since the COVID-19 outbreak has not been completely eradicated, we must be prepared for a great battle in the long term and for the regular management of the pandemic, which require doctors to work with great concentration to establish a strong defense against the pandemic, and it is an extraordinary test for doctors, both physically and mentally, in the long term.

To protect the physical and mental health of the doctors in charge of the regular management of the pandemic and to improve their joy in work, the government has published documents in succession. During the COVID-19 pandemic, the National Health Commission forwarded Measures to Improve the Working Conditions of Front-Line Doctors and to Care about Their Physical and Mental Health (2020) by the General Office of the State Council, the Ministry of Human Resources and Social Security, as well as the Ministry of Finance. The document pointed out that that moment was a critical time for national prevention and control of the COVID-19 pandemic and that doctors were faced with a heavy workload, high risk of high infection, inadequate working and resting conditions, and physical and mental pressure and other difficulties, and were prone to burnout, stressing the need to improve the working and resting conditions of doctors, the maintenance of their physical and mental health, and the strengthening of humanistic care for doctors (National Health Commission, 2020).

In this era of pandemic prevention and control, doctors, as the mainstay of health care, must assume greater responsibility and work. Moreover, it is vitally important to improve their health by improving their joy in work. Most existing studies judge overall joy in work based on scale scores, ignoring the heterogeneity of the doctor population (McDowell, 2004; Huang et al., 2010; Khanna et al., 2020).

Latent profile analysis (LPA) is an individual-centered approach to classifying samples based on different characteristics or variables of individuals and can identify inequalities in a population (Howard and Hoffman, 2018; Schmiege et al., 2018). Therefore, this study proposes to apply LPA to analyze joy in work among doctors in public hospitals, identify potential categories of joy in work and their differences, and provide a reference for the development of targeted interventions to enhance doctors’ work joy. In this study, we investigated the level of joy in the work of doctors in Chinese public hospitals and used LPA to identify doctors’ joy in work subtypes. The main objective was to identify doctors’ joy in work subtype characteristics and their associated factors based on the characteristics of questionnaire demographic variables. Previous studies have more often focused on the evaluation of joy in work or the negative direction of burnout and job stress measurement, which are important but do not delve into the differences between doctors’ joy in work. This study fills a gap in the literature and helps improve the understanding of public-hospital doctors’ joy in work and further identify the different subtypes of doctors’ joy in work that may be missing with the use of a single indicator.

## 2 Materials and methods

### 2.1 Participants

The sample of this study comprised of public hospital doctors involved in providing medical services during the COVID-19 pandemic. In addition, the target population was also regular hospital doctors who had been working in the hospital for over 5 months and were sufficiently familiar with the working environment situation in the hospital to ensure that pleasure was experienced while working in the hospital at the time; interns and graduate students or interns were not included in this study.

The sample size was calculated in three ways: first, the sample size based on reliability and validity should be 5–10 times or more than the number of items, therefore the number should be 140–280 or more; second, according to the formula $n=\frac{Z_{\alpha /2}^{2}P\left(1-P\right)}{\delta^{2}}$ (settings α = 0.05, *p* = 0.5, δ = 0.05), the sample size calculated by PASS 2021 software was 385 (Newcombe, 1998); third, the sample size should be more than 5–10 times the number of independent variables, which indicates 65–130 samples. In summary, at least 385 doctors were required for the study.

The study was carried out from January to May 2021 using a random sampling method. The questionnaires were distributed to doctors from public hospitals in Shanxi, Hubei, Guangdong, and Guangxi provinces in North, central and southern China, via a combination of online surveys (sojump, WeChat, etc.) and offline methods (field survey). In total, 426 surveys were distributed, and 426 valid surveys were returned, with a 100% efficiency rate.

### 2.2 Measures

#### 2.2.1 General information questionnaires

The subject group developed its joy in work measurement scale for doctors in public hospitals, which consists of four dimensions: work autonomy needs, competency identification needs, competency perception needs, and work relationship needs, with each containing seven items, resulting in 28 items. The scale was scored on a 5-point Likert scale with a possible score range of 28 to 140, with higher scores indicating higher joy in work for public school doctors. The reliability and validity of the scale were tested to be good, and the Cronbach coefficients of the total scale and four dimensions were 0.954, 0.872, 0.864, 0.892, and 0.912, respectively (Zhang et al., 2021).

#### 2.2.2 Statistical analysis

The data were analyzed using Stata 16.0 statistical software with statistical descriptions in terms of frequency, percentages, mean, and standard deviation, and the variability of demographic characteristics of potential categories of doctors’ joy in work was analyzed by chi-square test, followed by multivariate logistic regression for variables with significant differences at a test level of α = 0.05.

Using Mplus8.3 for LPA of doctors’ joy in work, the subscale scores of the four dimensions of work autonomy needs, competency identification needs, competency perception needs, and work relationship needs were used as exogenous variables, starting from one latent profile and increasing the number of latent profiles until the fit index of the model was optimal. The main evaluation metrics are Akaike’s information criterion (AIC), Bayesian information criterion (BIC), adjusted Bayesian information criterion (aBIC), Entropy, Lo–Mendell–Rubin likelihood ratio (LMR), and bootstrap likelihood ratio test (BLRT) (Gabriel et al., 2015). Akaike’s information criterion (AIC) is a standard for measuring the goodness of fit of statistical models and can balance the complexity of the estimated model with the goodness of fit of the data (Akaike, 1974). The Bayesian information criterion (BlC) is a criterion for model selection among a finite set of models, and models with lower BIC are generally preferred (Schwarz, 1978). The adjusted Bayesian information criterion (aBIC) is derived from the BIC by replacing sample size n in the equation that calculates the BIC with n* ((*n* + 2)/24), (Sclove, 1987). Lower AIC, BIC and aBIC values indicate better fitness of the data in the estimated model. LMR and BLRT compare the model fit between two adjacent models, and a significant *p* value indicates that the k-class model better suits the data than does the k-1 class model. Entropy evaluates classification accuracy, ranging from 0 to 1, where values closer to 1 denote improved classification, typically necessitating a threshold exceeding 0.8 (Gabriel et al., 2015).

## 3 Results

### 3.1 Participant characteristics

Among the 426 participating doctors, 63.18% were female, 92.02% were under or equal to the age of 45, and 58.22% were married. The majority of participants had an education level of undergraduate degrees or above (95.31%), held junior or middle professional titles (86.85%), and had 1–10 years of work experience (76.75%). Furthermore, 53.05% were from tertiary hospitals, and 80.04% were from comprehensive hospitals. The largest proportion held established positions (49.06%) and earned a monthly income ranging from 5,001 to 10,000 yuan (50.70%). In addition, more than half of the participants worked between 41 and 60 h per week (66.43%) and self-assessed their health as good (53.52%). The characteristics of the participating doctors are presented in Table 1.

**Table 1:** General characteristics of public hospital doctors (*n* = 426).

Variables	Number (%)	Variables	Number (%)
Gender		Hospital level	
Male	154 (36.15%)	Tertiary	226 (53.05%)
Female	272 (63.85%)	Secondary	161 (37.80%)
Age		Primary	39 (9.15%)
0–30	203 (47.65%)	Employment way	
31–45	189 (44.37%)	Establishment	209 (49.06%)
>45	34 (7.98%)	Personnel Agency	36 (8.45%)
Marital status		Contract	158 (37.09%)
Unmarried	172 (40.38%)	Others	23 (5.40%)
Married	248 (58.22%)	Department	
Divorced	5 (1.17%)	Internal	116 (27.23%)
Widowed	1 (0.23%)	Surgery	96 (22.54%)
Educational degree		Obstetrics	46 (10.80%)
Ph.D.	23 (5.40%)	Gynecology	31 (7.28%)
Master’s	96 (22.54%)	Others	137 (32.16%)
Bachelor’s	287 (67.37%)	Average monthly Income (RMB)	
Diploma or below	20 (4.69%)	0–5,000	126 (29.58%)
Professional title		5,001–10,000	216 (50.70%)
Senior	8 (1.88%)	10,001–15,000	55 (12.91%)
Deputy senior	48 (11.27%)	>15,000	29 (6.81%)
Intermediate	134 (31.45%)	Working hours per week	
Junior	236 (55.40%)	0–40	73 (17.14%)
Working years		41–60	283 (66.43%)
1–5	227 (53.28%)	>60	70 (16.43%)
6–10	100 (23.47%)	Self-rated health status	
11–15	45 (10.56%)	Poor	30 (7.04%)
>15	54 (12.68%)	Fair	168 (39.44%)
Hospital types		Good	228 (53.52%)
General hospitals	358 (84.04%)		
Specialized hospital	68 (15.96%)		

### 3.2 Potential categories of doctors’ joy in work

The indicators of each latent category of doctors’ joy in work are shown in Table 2. The values of AIC, BIC, and aBIC gradually become smaller as the number of profiles increases, but the difference in LMR is not significant when the number of profiles is 5; the value of Entropy is the largest when the number of profiles is 4, and the difference of LMR is statistically significant, but the probability of one of the categories is only 2.82% (<5%) without the practical significance of measurement (Wit et al., 2012). Considering the model parameters as well as the data inflection points, the model was best when the number of profiles was 3. The attribution probabilities of the three potential categories (Class 1, Class 2, and Class 3) were 94.30, 94.20, and 90.20%, respectively, indicating the high reliability of the results of the LPA.

**Table 2:** Latent profile analysis models and fit indices.

Model	AIC	BIC	aBIC	Entropy	LMR	BLRT	Proportion
1	10156.776	10189.211	10163.824				1
2	9678.576	9731.283	9690.030	0.872	0.389	0.000	16.90%/83.10%
3	9357.535	9430.515	9373.394	0.848	0.003	0.000	11.27%/59.86%/28.87%
4	9233.548	9326.800	9253.812	0.881	0.031	0.000	2.82%/60.33%/24.88%/11.97%
5	9171.440	9284.965	9196.110	0.828	0.070	0.000	2.35%/10.10%/42.72%/36.39%/8.45%

AIC, Akaike’s information criterion; BIC, Bayesian information criterion; aBIC, adjusted Bayesian information criterion; LMR, Lo–Mendell–Rubin likelihood ratio; BLRT, bootstrap likelihood ratio test.

**Figure 1:**
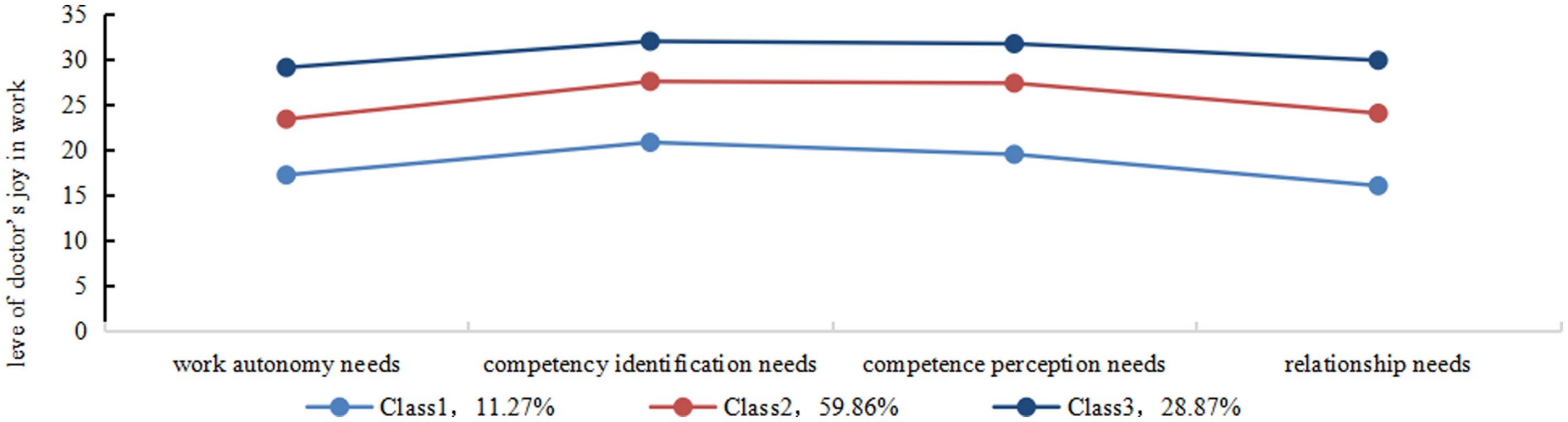
The latent profiles of the dimensions of the public hospital doctors’ joy in work evaluation scale.

The three categories of doctors’ joy in work on the four dimensions are shown in Figure 1. Class 1 that had lowest scores on all dimensions of joy in work was named the “low joy in work” category (*n* = 48, 11.27%), Class 2 that had a score between Class 1 and Class 3 was named the “medium joy in work” category (*n* = 255, 59.86%), and Class 3 with highest scores in all four dimensions was named “high joy in work.” category (*n* = 123, 28.87%). There were statistically significant differences in the scores on all dimensions of joy in work among three different types of doctors, further demonstrating the existence of within-group heterogeneity.

### 3.3 Analysis of job and demographic differences in latent categories of doctors’ joy in work

The chi-square test showed that the differences in the distribution of age, working years, hospital level, employment way, average monthly income (RMB), working hours per week, and self-rated health status were statistically significant (*p* < 0.05), while the distribution of gender, marital status, educational degree, and professional title was not statistically significant (*p* > 0.05). With the latent categories of doctors’ joy in work as the dependent variable, variables found to be statistically significant by chi-square test were included in the multinomial logistic regression analysis, and “high joy in work” served as the control group for deriving odds ratio coefficients to reflect the effects of the respective factors. The results of the multinomial logistics regression analysis are shown in Table 3.

**Table 3:** Multinomial logistic regression of doctors’ joy in work.

Variables	Medium joy in work				Low joy in work			
	*B*	*p*	OR	95%CI	*B*	*p*	OR	95%CI
*Age*								
31–45	0.228	0.535	1.255	0.612–2.575	−1.304	0.077	0.271	0.064–1.151
>45	1.930	**0.028**	6.886	1.237–38.340	−2.511	0.128	0.081	0.003–2.056
*Working years*								
6–10	−0.227	0.562	0.797	0.370–1.718	−0.251	0.755	0.778	0.160–3.772
11–15	0.485	0.394	1.625	0.532–4.959	1.753	0.073	5.773	0.848–39.305
>15	−0.913	0.204	0.401	0.098–1.642	1.923	0.084	6.842	0.772–60.631
*Hospital level*								
Secondary	0.973	**0.037**	2.646	1.061–6.596	1.754	0.080	5.776	0.811–41.168
Tertiary	1.288	**0.004**	3.625	1.493–8.804	2.416	**0.013**	11.199	1.678–74.735
*Employment way*								
Personnel agency	0.093	0.844	1.097	0.438–2.747	1.866	**0.021**	6.463	1.323–31.587
Contract	0.648	**0.035**	1.912	1.048–3.489	1.599	**0.006**	4.950	1.595–15.362
Others	0.821	0.213	2.272	0.624–8.277	1.936	0.082	6.934	0.783–61.363
*Average monthly Income(RMB)*								
5,001–10,000	−0.406	0.230	0.667	0.344–1.293	0.314	0.573	1.369	0.458–4.093
10,001–15,000	−0.945	**0.034**	0.389	0.163–0.929	−2.293	**0.040**	0.101	0.011–0.897
>15,000	−1.118	**0.050**	0.327	0.107–0.999	0.038	0.971	1.039	0.134–8.036
*Working hours per week*								
41–60	0.147	0.669	1.158	0.591–2.267	−1.124	0.067	0.325	0.097–1.083
>60	0.083	0.869	1.087	0.404–2.927	−0.312	0.676	0.732	0.170–3.151
*Self-rated health status*								
Fair	1.020	0.286	2.772	0.426–18.039	−2.403	0.018	0.090	0.124–0.660
Good	−1.350	0.146	0.259	0.042–1.601	−6.151	**0.000**	0.002	0.000–0.018

OR, Odds ratio; 95%CI: 95% confidence intervals.

*p* values reaching statistically significant (*p* < =0.05) are shown in bold.

*p* values are obtained from the *F*-test for continuous variables and the *Χ*^2^ test for categorical variables.

The results showed that the risk factors for the “medium joy in work,” using the “high joy in work” as a reference, were age more than 45 years (OR = 6.886, 95% CI: 1.237–38.340), working in secondary or tertiary hospitals (OR = 2.646, 95% CI: 1.061–6.596; OR = 3.625, 95% CI: 1.493–8.804), and being on contract (OR = 1.912, 95% CI: 1.048–3.489), and the protective factors were average monthly income of 10,001–15,000 yuan (OR = 0.389, 95% CI: 0.163–0.929); risk factors for “low joy in work” were tertiary hospital work (OR = 11.199, 95% CI: 1.678–74.735), personnel agency (OR = 6.463, 95% CI: 1.323–31.587), and being on contract (OR = 4.950, 95% CI: 1.595–15.362), protected by the average monthly income of 10,001–15,000 yuan (OR = 0.101, 95% CI: 0.011–0.897), and fair self-rated health and good self-rated health (OR = 0.090, 95% CI: 0.124–0.660; OR = 0.002, 95% CI: 0.000–0.018).

## 4 Discussion

### 4.1 Need to improve doctors’ joy in work

This study identified three categories of doctors’ joy in work. The total percentage of doctors with medium and low joy in work is over 70%, which indicates that most public hospital doctors’ joy in work needs improvement. Doctors with “low joy in work” have the lowest degree of satisfaction with work autonomy needs, competency identification needs, competency perception needs, and work relationship needs. According to self-determination theory, deprivation and blockage of basic psychological needs inhibit the full potential and self-actualization of individuals and even lead to maladjustment or mental illness (Deci, 1971; Deci and Ryan, 1980, 1985). A study confirmed that satisfaction with basic psychological needs was found to be negatively related to negative work correlates. When the satisfaction of basic psychological needs of doctors is low, their work engagement will decrease and they are more likely to develop burnout (Gagné et al., 2015). Therefore, hospital managers should focus on this type of doctor and give them timely care and assistance to enhance their joy in work. It is reasonable for hospital managers to organize doctors’ consulting shifts and guarantee their time off in order to enable them to enjoy both work and rest and to improve their work autonomy and competency needs. In addition, medical institutions can also optimize workflow and adjust doctors’ work tasks by advocating medical and nursing cooperation as well as multidisciplinary teamwork, creating additional diagnostic and treatment support positions such as doctor assistants, strengthening information technology, and providing lean management such that doctors have the time and energy to focus on patients themselves and the diagnosis of their conditions, provide high-value medical services, and improve job competence and fulfillment (Plant et al., 2016; Zhang et al., 2020).

### 4.2 Characterization of doctors in medium joy in work categories

A better understanding of the differences in demographic characteristics among doctors in different joy in work categories can help hospital administrators with early identification and precise intervention. The study results showed that hospital level, employment way, and average monthly income (RMB) had a significant effect on doctors’ joy in work. The reason is that tertiary hospital doctors are more likely than primary hospital doctors to belong to the low and medium joy in work category, which is probably because tertiary hospitals are equipped with superior facilities and technology, receive more difficult and complicated patients, have a high volume of consultations, and have a complicated caseload; in these hospitals, doctors are in a state of high demand and high workload for a long time and their work autonomy and competence are poor (Wen et al., 2016; Li et al., 2022). Furthermore, as the primary provider of medical treatment during the pandemic, doctors in tertiary hospitals have to bear the pressure of treating patients and also face the risk of being infected. Moreover, their workloads are heavy with tedious tasks and without much joy in their work (Peisah et al., 2009).

Doctors who were older than 45 years, on contract, and working in a secondary or tertiary hospital were more likely to have “medium joy in work” than “high joy in work.” Hospital level and doctor’s age influence patients’ choice of care, with higher-level and older doctors being preferred by patients due to more training and practical experience, as well as experience with a higher number of outpatients and inpatients. Lower levels of burnout, greater job satisfaction, and higher joy in work are experienced as age and seniority increase (Dırvar et al., 2021). However, since older doctors generally have higher seniority and positions and have to undertake more clinical and managerial work in the departments, they are under a high state of mental strain. Meanwhile, increasing age can also result in impaired physical function, which, in turn, weakens their level of joy in work and ultimately results in them belonging to the “medium joy in work” category. Interestingly, in contrast to previous studies, doctors aged 0–30 years were just entering their new job, were enthusiastic about their work, and were usually taught by clinical teachers, and their clinical decision-making was not overly stressful and not less joyful. Doctors with poor self-rated health tend to feel a lack of stamina and energy when confronted with high, long working hours and almost zero-error standards of clinical work. These high standards can isolate individuals who make mistakes, leaving them without a healthy coping style, which, in turn, leads to a dysfunctional approach to recovery and a greater risk of burnout. This results in a subsequent decrease in competency identification, and competency perception (Wu, 2000; Tamburri, 2017). In addition, the higher the level of hospitals and the more functions it undertakes, the greater the demands on the technical capacity and specifications of doctors’ treatments. Not only do doctors have to complete a large amount of diagnosis and treatment of the disease, but they must also spend time completing case registration and management, health promotion, and research and teaching, resulting in poor competency identification and a sense of competence perception in their work due to their long-term high demands and workload (Zhang and Feng, 2011).

Contract doctors are worse than established doctors in terms of workload, career development, compensation and benefits, and retirement benefits, and these doctors do not have a sense of stability and belonging and lack sufficient work enthusiasm and concentration; as such, they do not have much joy in work (Wang and Lin, 2015; Li et al., 2020).

The average monthly income of RMB 10,001–15,000 is its protective factor. It can be seen that economic factors are important factors that affect doctors’ joy in work, and income is not only a basic guarantee of life for doctors but also a full expression of their self-worth, and higher income can bring more competent identification and perception. Therefore, it is necessary to increase doctors’ pay-for-performance income through a variety of effective incentives and, thus, improve the overall level of income and increase their motivation to work (Cai et al., 2022).

### 4.3 Characterization of doctors in low joy in work categories

Compared with “high joy in work” doctors, “low joy in work” doctors are mainly on personnel agency or contract, work in tertiary hospitals, have an average monthly income of 10,001–15,000 RMB, and have good self-assessed health status as their protective factors. It is of concern that personnel agency doctors do not have high joy in work. A study showed that hospital personnel agency have problems such as poor social security, low identity, and limited space for career progression (Chen, 2017). Although equal pay for equal work has been implemented in some hospitals, a lack of supportive safety still affects doctors’ sense of community and professional identity.

In addition, fair or good self-rated health status is another protective factor for “low joy in work” doctors. Individuals who can maintain good health in groups that experience substantial life stress have greater resilience to stress and the negative effects of burnout (Folkman, 1997; Folkman and Moskowitz, 2000; Moskowitz et al., 2017, 2019). Personnel with good self-rated health had higher psychological resilience, which could help them to adapt to their work tasks and recover more easily in the face of negative events and emotions (Saragih et al., 2021; Prakash et al., 2023). However, several studies have shown that healthcare workers have poor self-rated health status, thus necessitating measures to enhance their health status. First, regular health check-ups and mental health knowledge training are organized, and recreational activities are conducted to enhance colleagues’ communication so that doctors can feel support from the organization and colleagues. By introducing programs such as the Employee Assistance Program, work and emotional support are provided to doctors who are professionally maladjusted, increasing their resiliency and work competence, and so on. Second, doctors with “high joy in work” are full of enthusiasm for their work, can recognize the meaning and sense of achievement in their work, and manage their work independently and flexibly; thus, they can be developed into peer mentors, and through one-to-one support and experience sharing, they can drive doctors with “medium joy in work” and “low joy in work” to learn self-adjustment.

## 5 Limitations

This study has several limitations. First, the outcome indicators used in this study were obtained through self-reporting, which may be biased. Second, this study was a cross-sectional survey, and the sample size was small, and the OR value may be too large or too small due to the influence; longitudinal follow-up surveys and expansion of the sample size could be considered in the future. Third, this study did not collect relevant data to evaluate the factors that may be protective factors through psychotherapy and counseling interventions, further study can focus on these intervention means to improve the joy in work of doctors.

## 6 Conclusion

The study aimed to explore the potential categories and characteristics of doctors’ joy in work among Chinese public hospital doctors, using latent profile analysis. The findings revealed three potential types of joy in work, which were low, medium, and high. Additionally, the study used multiple logistic regression analysis to identify factors contributing to low joy in work, finding that personnel agency and contract doctors in tertiary hospitals were more likely to have low joy in work than other types of doctors. Furthermore, the study identified protective factors for low joy in work, including having an average monthly income of RMB 10,000–15,000 and fair or good self-rated health status. These findings suggest that improving doctors’ joy in work could be achieved by improving their income and supporting their physical and mental health. Overall, the study provides valuable insights into the potential categories and characteristics of doctors’ joy in work and highlights the importance of addressing specific factors, such as income and health, to improve doctors’ work experience. These findings can inform hospital managers and policymakers on ways to enhance job satisfaction among doctors in public hospitals.

## Data availability statement

The raw data supporting the conclusions of this article will be made available by the authors, without undue reservation.

## Ethics statement

Ethical review and approval was not required for the study on human participants in accordance with the local legislation and institutional requirements. Written informed consent from the patients/participants or patients/participants’ legal guardian/next of kin was not required to participate in this study in accordance with the national legislation and the institutional requirements.

## Author contributions

WZ: Data curation, Investigation, Methodology, Software, Writing – original draft, Writing – review & editing. JL: Software, Writing – review & editing. LW: Writing – review & editing. FD: Writing – review & editing. YZ: Writing – review & editing. KD: Writing – review & editing. HZ: Writing – review & editing.
